# The feasibility of a randomised controlled trial of Dyadic Developmental Psychotherapy

**DOI:** 10.1186/s12888-014-0347-z

**Published:** 2014-12-30

**Authors:** Fiona Turner-Halliday, Nicholas Watson, Nicole RS Boyer, Kathleen A Boyd, Helen Minnis

**Affiliations:** Institute of Health & Wellbeing, University of Glasgow, Academic Unit of Mental Health & Wellbeing, Caledonia House, Royal Hospital for Sick Children, Yorkhill, Glasgow G3 8SJ, UK; Institute of Health & Wellbeing, University of Glasgow, College of Medical, Veterinary and Life Sciences, 1 Lilybank Gardens,, Glasgow, G12 8RZ UK; Institute of Health & Wellbeing, University of Glasgow, Health Economics and Health Technology Assessment, 1 Lilybank Gardens, Glasgow, G12 8RZ UK

**Keywords:** Dyadic Developmental Psychotherapy, Feasibility, Evidence, RCT, Qualitative, Measurement

## Abstract

**Background:**

Maltreated children have significant and complex problems which clinicians find difficult to diagnose and treat. Previous US pilot work suggests that Dyadic Developmental Psychotherapy (DDP) may be effective; however, rigorous evidence from a randomised controlled trial (RCT) is lacking. The purpose of this study is to establish the feasibility of an RCT of DDP by exploring the ways that DDP is operating across different UK sites and the impacts of current practice on the potential set-up of an RCT.

**Methods:**

Qualitative methods (interviews, focus groups and teleconferences) were used to explore trial feasibility with therapists and service managers from teams implementing both DDP and possible control interventions. Data were analysed thematically and related to various aspects of trial design.

**Results:**

DDP was commonly regarded as having a particular congruence with the complexity of maltreatment-associated problems and a common operating model of DDP was evident across sites. A single control therapy was harder to establish, however, and it is likely to be a non-specific and context-dependent intervention/s offered within mainstream Child and Adolescent Mental Health Services (CAMHS). Because a ‘gold standard’ Treatment as Usual (TAU) does not currently exist, randomisation between DDP and TAU (CAMHS) therefore looks feasible and ethical.

The nature of family change during DDP was regarded as multi-faceted, non-linear and relationship-based. Assessment tools need to be carefully considered in terms of their ability to capture change that covers both individual child and family-based functioning.

**Conclusions:**

An RCT of DDP is feasible and timely. This study has demonstrated widespread interest, support and engagement regarding an RCT and permissions have been gained from sites that have shown readiness to participate. As maltreated children are among the most vulnerable in society, and as there are currently no treatments with RCT evidence, such a trial would be a major advance in the field.

## Background

Children who have experienced maltreatment through abuse and neglect in early life are at greatly increased risk of significant mental health problems [1,2] which are complex, difficult to classify and can include Reactive Attachment Disorder (RAD), conduct problems and Attention-Deficit Hyperactivity Disorder (ADHD) [2-6]. For the purposes of this project, we therefore refer to the problems resulting from early abuse and/or neglect as “maltreatment associated psychiatric problems” (MAPP). Whilst MAPP can have very significant and burdensome effects [7], many children are not routinely offered CAMH services [8] and, even where they are, there is evidence to suggest that they languish on the caseloads of CAMHS clinicians because effective treatments are unavailable [9].

Dyadic Developmental Psychotherapy (DDP) is the only intervention that we are aware of that incorporates all of the elements suggested by the literature for the treatment of MAPP [10,11]. It is based on the premise that child development is dependent upon, and highly influenced by, the nature of the parent–child relationship and that development of this relationship requires ongoing, dyadic (reciprocal) experiences between parent and child. DDP uses core principles of playfulness, acceptance, curiosity, and empathy (commonly referred to as ‘PACE’) in order for the parent and child to “co-create” new meaning regarding past experiences [11].

DDP is an intensive therapy offered, usually, over several months. The human resources required to deliver DDP are considerable (approximately 20 1–2 hour sessions), but may well be outweighed by the benefits especially when balanced against the burden of these children’s problems on the NHS and costs to society of not treating MAPP [8-13]. Previous work conducted in the United States suggests that DDP has a large effect size when offered to maltreated children with MAPP [14] and those receiving DDP improved significantly over time, while those receiving other therapies deteriorated [15]. Whilst the US studies are reasonably robust, they are not definitive; they are based on the results of comparing children having DDP with a matched control group of similar children having a range of other psychotherapeutic interventions, but the DDP was conducted by a single therapist and was not randomised. This means that, despite these promising findings to date, randomised controlled trial (RCT) evidence, which is regarded as the highest level of evidence in terms of quality [16], is currently lacking for DDP.

RCTs of complex interventions, particularly in social work contexts, are notoriously challenging given the multiple aspects of variation and difficulty in achieving standardisation [17]. It is therefore important to learn from the few trials with maltreated children that have gone before. One such randomised controlled trial [18] – and the researchers’ careful reflection upon it [19] – demonstrated the need for very deliberate enquiry with a multi-centre design. The environment for that trial was particularly challenging as there is less of a history of randomised trials recruiting from within local authority social work, whereas recruiting for similar studies within the NHS has generally been much more successful e.g. [20].

Given the established need for an RCT of DDP, this qualitative study was set up to explore practices regarding the therapy in various centres across the UK in order to understand more about the model of DDP across different contexts and the factors that impact on its design and delivery. Overall, we aimed to establish the feasibility of, and optimise conditions for, a multi-centre RCT investigating the effectiveness and cost-effectiveness of DDP as a treatment for maltreated children with MAPP when delivered in various UK NHS centres.

The primary research question was: Is a multicentre RCT of DDP versus a control intervention feasible?

Subsidiary questions were focused on: 1)The ways in which DDP was practiced across UK DDP sites, probing practical aspects of delivery (e.g. duration and frequency of treatment), rationales for the models adopted and contextual factors affecting the use and delivery of DDP; 2) Perspectives on how an RCT would work within sites, exploring views on randomisation, whether a potential control intervention existed within sites, measurement of change and how sites define their eligible population for DDP.

## Methods

### Design

This study has followed a three-stage process of data collection using a range of qualitative methods including semi-structured telephone interviews, focus groups and teleconferences with sites across the UK that had previously reported use of DDP during networking at a UK conference, or had been reported to us by UK DDP trainers. Our local NHS ethics committee (Greater Glasgow and Clyde Research and Development Department) confirmed that ethical approval was not required for the study, given that the participants were all professionals.

Each stage of data collection informed the next, following an iterative approach that a) allowed the study to evolve in relation to the issues being unearthed and b) to be responsive in relation to changes within sites or new sites becoming visible during the process of the study. Some sites that had initially shown interest during networking were not followed up after initial scoping work (i.e. telephone calls to managers) revealed changes in circumstances since first contact (e.g. DDP no longer being funded). This left eight main areas and these have made up our key DDP sites for formal data collection.

### Data collection

The three stage process of data collection can be described as a ‘funneling’ approach from the exploration of general models (across sites) to investigating more specific aspects of process and context within sites. The stages of this process were the following:

1. Thirteen semi-structured telephone interviews were carried out with DDP therapists and managers. These allowed us to explore the ways in which the therapists conceptualise and practiced DDP within each the eight sites. Economic data regarding the NHS and social services resource use in delivery of DDP services was also gathered at this stage; the findings and costs of the services are presented in an accompanying paper [11].
2. Focus groups were conducted in four out of the eight sites that we initially identified and explored through the interviews. Of the four sites not followed-up after the interviews, three sites reported that DDP had become compromised because of funding and there was significant uncertainty over its immediate future and one site declined further involvement in the study. Since many changes were apparent within sites throughout the study, however, these sites were not ‘excluded’ from a potential trial; rather, it was decided that they would form a “secondary site list” to be re-visited should an RCT be designed. Indeed, one site from this list went on to obtain significant funding for DDP during the course of the study and therefore re-entered the study.
  The focus groups were primarily focused on how a trial might work within the context of the particular sites involved. They also allowed us to further explore themes and seek clarification about any issues that had emerged at the interview stage.
3. Teleconferences (due to geographical distance) or follow-up meetings (for those more local) were conducted with the remaining five sites. This allowed follow-up of agreements and negotiations made after focus group discussion and also allowed inclusion of a further participant who had been identified as key stakeholder during the focus group stage. This follow-up stage also allowed the research team to propose a likely trial model to the sites after analysing the data already gathered. In this sense, the teleconferences and meetings provided an opportunity for bi-directional feedback allowing the research team to update therapists and managers in the sites about methodological thinking whilst gathering further data about progress since the focus groups.
  Attendance at a UK DDP conference (Manchester, June 2012) was also an opportunity for data collection, networking and presentation of interview findings including a video-recorded discussion and debate about key issues in relation to a potential trial.

### Data analysis

All data gathered (on phone, face-to-face and teleconference) was recorded and transcribed verbatim. Identifying information was removed from transcripts to anonymise the data and transcripts were stored securely and treated confidentially. The data was analysed thematically, organising participants’ responses into common threads of perception and relating them to the design of an RCT.

All stages of methodological decision-making, from design to analysis were considered in relation to quality guidelines for qualitative research, which meet the RATS (qualitative research review) guidelines focusing on relevance, appropriateness, transparency and soundness of the interpretive approach [21]. The researcher who carried out the data collection and analysis for this study is a non-clinical health psychologist with post-doctoral expertise in qualitative research.

## Findings & discussion

Themes are organised into three main categories. The first details the findings that emerged in relation to DDP in practice across the different contexts. This theme addresses a central question about whether there is sufficient similarity amongst variable DDP models, both in terms of practical factors and conceptual thinking about DDP. The second main theme unpacks some of the thinking around specific aspects of RCT feasibility; whether randomisation would work and the ways in which eligibility for DDP might be defined. (What do we compare DDP against and how do we measure change?) examines what might constitute a feasible control in an RCT of DDP and therapists reflections on the most important aspects of progress in DDP, impacting on the utilisation of assessment measures in an RCT.

## DDP in practice: how is it used in the UK and why is it suitable for MAPP?

### Models of DDP

DDP was described as being used in two different ways across sites; first, in its classic sense as a therapeutic model (often described as ‘full’ DDP) and, second, as a model that provides guiding principles in terms of general interactions between therapists and families, but also between team members in DDP contexts and in training contexts. The principles of PACE were seen as pivotal to providing a philosophical way of working that led some therapists to report that DDP “permeated” the nature of their whole service:

*We do training with foster carers as well in both Local Authorities and, you know, the principles of DDP are kind of peppered through that as well. We talk a lot about PACE and what kind of response the children they are looking after would need, and what we think might be helpful. So when you mention Dan Hughes* [founder of DDP] *you are not seeing a foster carer going ‘who?’ His work is very alive. (Site B)*

If an RCT is to be rolled out, it is clear that the model of DDP will have to be closely monitored. Most of those we spoke to felt that the full version of DDP should form the basis of any planned trial:

*I think, you know, for creditability in terms of a RCT we probably need to go for this ‘full-fat’ model…almost manualised (Site F).*

**Figure 1:**
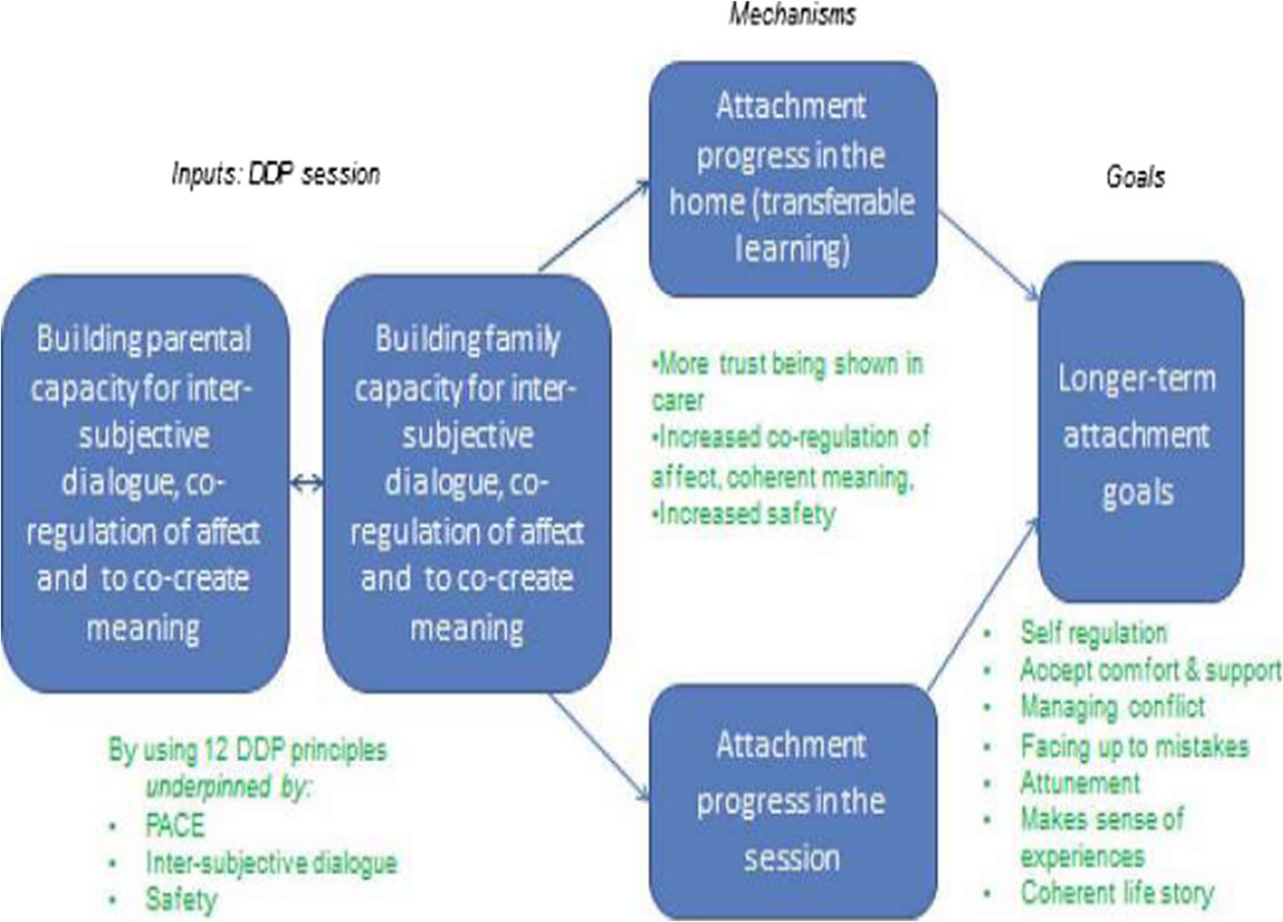
**DDP logic model (Logic model developed for the workings of DDP from interview data with DDP therapists and feedback from DDP experts).**

Across the first stage of data collection (interviews), the mechanics of the ‘full fat’ model of DDP were reflected upon, and we used these data to construct a logic model. An early draft of this was presented at a UK DDP conference and scrutinised by world-wide experts in DDP (including the founder of DDP – Dan Hughes). Many of the research participants attended the conference, giving the opportunity for further reflection on our interpretation of the ways in which DDP works. The logic model was refined after the conference according to feedback (see Figure 1).

In addition to exploring the mechanisms by which DDP operates, practical factors such as the typical duration and frequency of DDP formed part of the interview questions and there was a common pattern found across sites. Also overarching all interviews was a general consensus that the DDP model has to fit with the complex and changeable circumstances of MAPP and care situations:

*There are cases that I kept on because of particular crisis points in a placement for the child so it* [duration of DDP] *is very dependent on lots of variables. (Site B)*

The ability of DDP to be responsive and adaptable was seen as a key strength of the model; however, for the purposes of designing an RCT, careful consideration needs to be given to the duration of the assessment phase and the time points of measurement and follow-up.

### The suitability of DDP for MAPP

In addition to being able to identify shared models of DDP across sites, there was significant evidence of there being a common philosophical understanding of DDP in terms of its suitability to address MAPP. Four common themes emerged from the data in relation to this issue:

#### DDP as a fit with the multidimensional complexity of MAPP

The complexity of the problems faced by looked after and adopted children and their families was a major theme in the data. This, according to our participants, meant that many therapeutic approaches were unable to meet needs in relation to the multifaceted and interacting nature of the children’s problems:

*The dilemma for the looked after children really is that what’s brought them into the situation is very complex. So a lot of the NICE research of course is based on single issues like ‘well what do you do about depression?’ … There are all sorts of complex things that get in to it (Site E).*

Most therapists felt that the multi-faceted nature of the problems these children faced meant that only a multi-dimensional therapy such as DDP was suitable. As the next sub-theme captures, therapists also felt that the complexity of MAPP was matched by DDP’s relationship-based and external focus.

#### A relationship-focus on the external world of the child

Participants described how DDP allows the complexity of problems to be addressed by maintaining the focus of therapy on the external relationship-based world of the child. DDP allowed the therapists to ‘stay true to’ the origins of maltreatment as being externally experienced:

*The main advantage for me is that it takes the focus away from the child as being the problem and by including the adult in the therapy it brings it back to the situation and the environment the child is in. The way I explain the approach that we take is this: If it was the environment that damaged these children; the neglect, the abuse, you know, multiple rejections etcetera, then it is the environment that has to fix it (Site H).*

Including the carer/parent in therapy was seen to promote shared ownership of MAPP with parents/carers acting as co-therapists. This was seen as key to long-term maintenance of change:

*I think the thing about DDP is that it makes it very explicit right from the very start that the parents and carers are the primary agents of change really and that is a kind of mind-shift there…change doesn’t happen for the child in a vacuum and it is much more successful if it is in a strong relationship (Site B).*

Other therapies that are designed for children without the presence of the carer/parent were seen as conveying an unrealistic and detrimental message to children with MAPP that responsibility for progress is self-driven:

*I think the difficulty I have with other therapies for these children is that if you have a therapy that brings a child into the appointment and the adults stay outside in the waiting room or go shopping or something and come back to pick them up, it puts a huge pressure on a child to be fixed; in other words, it gives that very clear message that the solution is within them … Now that I would see as quite dangerous because it compounds that belief that they have of themselves that they have got the problem (Site H).*

#### ‘Current moment’ interaction between past and present

DDP’s focus on what is happening in the room between child and carer/parent during the therapeutic intervention is seen as a key mechanism of the therapy. It allows the therapist to address “clashing” external realities between past and present with the capacity for change resulting from the ability to observe and direct the relationship between child and carer as it is happening:

*When you are dealing with new relationships, you need that relationship in the room…because that’s the key to the change and to healing really. Whereas when you are doing non-directive play therapy or psychotherapy in a mainstream situation then you are working with the internal world of a child whereas this is about working with the internal world of the child which is driven very much by extreme external experiences in their past, which are clashing with their current external experiences. So it is different…in the room you talk out loud with the child and the parent about what’s happening between them. It gives you an opportunity to reflect and problem solve in a sense, but in real time (Site C).*

This ‘in the moment’ focus was seen as directive in terms of the therapist’s input; a point that was seen as an essential difference to other forms of therapy:

*We do things like seeking to have the parent talk direct to the child, or the child talk direct to the parent, telling the parent how they are feeling and if they can’t do it then we, the therapist, with the child’s permission, will talk for the child and then the parent will answer. So you deepen the interaction by getting the conversation happening in the room (Site C).*

These techniques allowed a “co-creation of new meaning”, allowing the present relationships in the room to collaboratively create meaning about the past. Linking to the previous theme, the focus on the relationship, rather than the child, is the facet that allows a shared sense of growth to occur:

*Dan Hughes talks about co-creating two things; co-regulating emotion because these children tend to be very emotionally un-regulated and also co-creating meaning, so that the meaning for the child of who they are and what’s happened to them is co-created by this new set of people. So whereas they might have perceived their early life as what they deserved, by taking them back there emotionally as well as cognitively, and sharing it with them, we create a new meaning for the child of what happened to them (Site C).*

#### The capacity for change

Whereas attachment theory had allowed therapists an understanding of the relational issues that children with MAPP present with, therapists reflected that DDP provided therapeutic tools that could actually change attachments:

*We have a theoretical understanding of the impact of their past experiences on their relationships, but the big question then is ‘okay so how do we make it any better?’ And I suppose it [DDP] was the first time that I had come across concepts and approaches that gave you a ‘what to do next?’ rather than just assess about attachment (Site C).*

Essentially, DDP gave therapists what they felt was a way forward for a group of children for whom other treatment approaches were often inadequate:

*I think the difference was, and this is a difference within not just my practice but also within the wider field of theory, is how the hell do you make it applicable moving from assessment into treatment? That’s a big step, and I think that was the bit for me that Dan did - suddenly I could take all this knowledge I had about it, assessing children, and actually make it into something that can actually work for the child; to make sense of and to work through. The light went on and now I see a way forward (Site F).*

## Who is most suitable for DDP? Defining the eligible population

The study aimed to define the population of children for whom DDP is most suitable and hence determine criteria for inclusion in a trial of DDP and explore the feasibility of randomisation between therapies. Two main themes emerged in relation to this research question; first, there is a conceptual ‘blurring of boundaries’ between children deemed as suitable for MAPP in comparison with other therapies; second, DDP requires a sense of readiness and, often, work to enhance readiness before the child and parent/carer can begin DDP together in the same room.

### Blurred boundaries between target populations

Looked after and adopted children have multiple and complex needs and participants in the study reflected on the blurred boundaries between populations that they have deemed suitable for DDP or potential control interventions. Whereas some participants saw distinctions between these groups, most therapists reported that those most eligible for DDP were children with MAPP for whom relationship-based problems were at the forefront of their difficulties; however, these children could still have traditionally diagnosable mental health problems. It was felt that MAPP could be read as either “psychiatric” or “relational” with perceived differences reflecting clinical judgement rather than actual separable issues:

*So one set of people assessing a child would give them a psychiatric diagnosis and another one would say that it is adoption issues; it is not psychiatric. It becomes a bit about semantics, to be honest (Site C).*

*A partial group within looked after can almost be randomly allocated to a diagnosis of ADHD or not a diagnosis of ADHD depending on what paediatrician they were allocated to and similarly for ASD, so it starts to look extremely random (Site E).*

Participants also surmised that distinctions made between diagnoses or eligibility can often be made on the basis of capacity issues and the presence of specific therapists at the time of assessment:

*Clinicians who might start off the process might have a view that ‘x’ would be helpful so they will look at things from their own therapeutic perspective (Site B).*

Resource and capacity often affected the therapeutic journey that a child experienced and resulted in the child being treated through a variety of approaches prior to DDP until a therapist was free to take on the work:

*There was a boy of twelve a few months ago who came in and it so happened that at that particular point the only therapist we could offer was a CBT therapist, so she went and worked with him and it was hard work - he found it hard work and she found it hard work -but after four sessions she came back saying to me ‘this guy needs play therapy, he is not managing to engage with the cognitive stuff at all’. We had a really close look at it and actually it is relationship repair work with his mother that needs done. So I would be arguing that’s a case that DDP would be the appropriate approach. Now as it turns out we don’t have anyone available at the moment that can take that case on (Site B).*

DDP therapists often worked closely and collaboratively with other therapists in mental health services and provided joint therapies for MAPP, with therapeutic journeys reflecting instances where children with MAPP moved between DDP and other psychiatric treatments depending on their need:

*We’ve had a young lady who was refusing to eat and she really needed proper psychiatric work but part of the reason why this was happening was because of a lot of insecurity in the relationship so it seemed complementary to be able to offer DDP when CAMHS were saying ‘we can’t do that relational work.’ It is about recognising that one approach doesn’t fit all really (Site C).*

It was also notable that those who initially talked about distinctions between children receiving DDP compared to other interventions would often start to question, later in the process, whether there was actually a difference between the groups in terms of the problems that they faced. Subtle shifts in thinking were evident, particularly in focus group contexts where discussion and debate can change individual opinion during the research process [22].

### The issue of ‘readiness’

Whereas all looked after and accommodated children experiencing MAPP were seen as the eligible population for DDP, a second main theme to emerge from the data in relation to inclusion criteria was that the full model of DDP requires a state of readiness. For a minority, it was the child who had to be ready whilst for others it was the carer/parent. Where this speculation related to the child, it was made in reference to the need to carry out prior work with the child before commencing DDP. In this perspective, differentiating between DDP and a different therapy can also be made on the basis of whether a child is *ready* for DDP rather than whether it is fundamentally suitable for them:

*There were a couple of cases recently whereby I had a dilemma as to whether or not to offer DDP or whether to offer psychotherapy, and actually on both of them I’ve offered psychotherapy.... well I have referred them on for psychotherapy, that’s with the view to them coming back to DDP. And I think, for me, it’s something about the presentation of the child, whereby their sense of themselves is not integrated (Site E).*

The majority of therapists, however, felt that readiness was mostly in relation to the ability of carers/parents to create a safe and nurturing environment within which DDP can operate. Carers/parents have to be able to act as co-therapists and respond appropriately to the child in joint sessions in a way that is congruent with PACE principles. This can be ongoing throughout work with a family, particularly at times where it becomes apparent that the carer requires individual support or when their reactions to the child become incongruent with a DDP approach:

*I am working with a couple of experienced foster carers who are kind of skirting over a child’s anxiety using humour and they don’t realise they are doing it. Talking to these adults you hit a brick wall….they are not seeing things but when I notice it in the session and draw it to their attention I am hoping they can start to notice it in their day-to-day life. For me that is one of the main benefits of the actual treatment; you’re doing that feedback in the room with them. They can’t do it on their own (Site A).*

While some saw readiness work as a ‘lead-in’ to DDP, others saw this work as an integral part of a DDP model:

*I would always start off by doing some work with the carers first because you need to know that they are not going to abandon a child emotionally in a session and that the child is going to be supported. So actually the full model would include the fact that you might have to work with the parents for a considerable length of time (Site B).*

In some sites, the readiness work with carers was amalgamated with other therapies that involved observing the child and carer together. For example, in one site, play therapy was often used as a starting point for assessing attunement in the relationship:

*If I play some attachment based games with them together, I come out with a very clear idea usually of where they are at and whether they are able to attune with each other (Site B).*

Other therapists saw DDP as a first line approach that can be followed by other therapeutic approaches:

*I think that in a way I would see DDP as the obvious first step kind of approach and it might be that through a period of skilling up the foster carers, and helping the child communicate some of these core difficulties and reasons why they are finding it so hard to be with these carers, that would open up the possibility of accessing other sorts of therapy (Site A).*

The findings in relation to work with carers within a DDP model evoke questions for an RCT about when baseline measurement should start. In one site, this issue was discussed in a phase three teleconference and it was felt that if readiness work with carers is an integral part of a DDP model, rather than classed as additional ‘lead-in’ work, then baseline measurement should be implemented before readiness work commences.

The findings also suggest that a trial model should reflect the fact that the eligible population of children ready to start DDP could become narrower after an initial assessment of carer readiness by sites, but that there would be potential within the lifetime of the trial for some families to progress to a stage of readiness to commence this stage of DDP. The data also suggests that the model should reflect the fact that there might be instances where work to enhance readiness is ineffective and DDP remains unsuitable for the lifetime of the trial:

*I suppose you might be thinking about the DDP process starting at the point where you decide you are going to see whether the carers are up for it, so you might have a drop- out rate because you decide, ultimately having worked with carers for a few sessions, that actually this isn’t on, we will have to try something else (Site G).*

**Figure 2:**
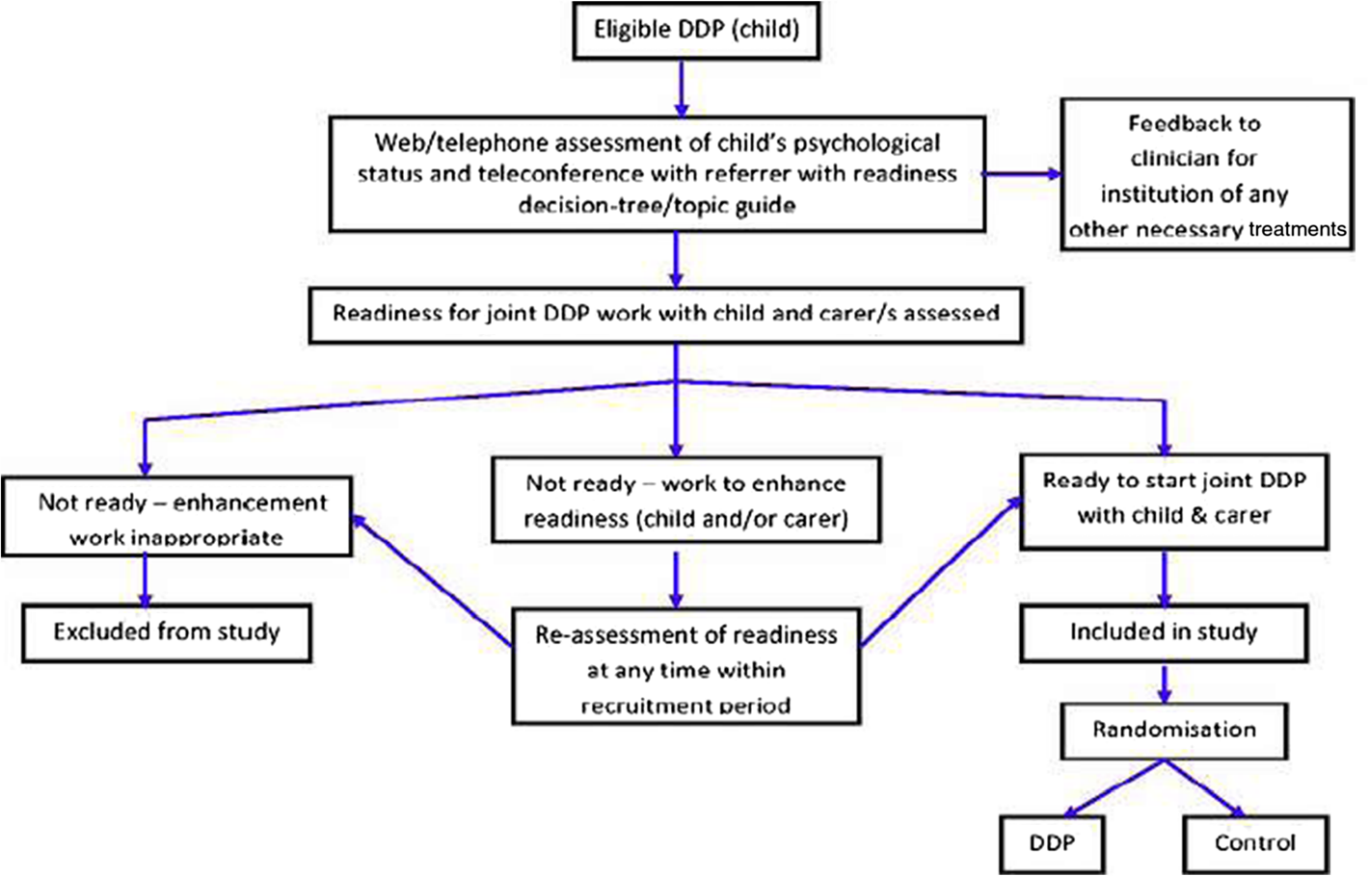
**Trial model (Exploratory trial model delineating the process of randomisation between DDP and the control intervention in an RCT).**

Our proposed trial model therefore allows for readiness variability and changeability. In light of the findings of this study, we propose that ‘readiness’ work be seen as part of the DDP model – as a first stage and as ongoing work throughout the therapy. These pathways are teased out and illustrated in the trial model (Figure 2).

## What do we compare DDP against and how do we measure change?

Within the wider context of services in each site, we explored the nature of other therapies that children with MAPP were offered. It was hoped that this would give insight into a likely control intervention in terms of Treatment as Usual (TAU) when DDP is not available or appropriate. Therapists’ views on how DDP should be measured were also gathered. The following two themes represent participants’ perspectives in relation to these two trial-related concepts:

### The variability and complexity of Treatment as Usual

Whereas this study has gained in-depth insights about the ways in which DDP is being implemented across different contexts, we have only scratched the surface in terms of what TAU might look like in each area for the purposes of a control intervention. Sites tended to be unable to identify a specific intervention or service that they could definitively class as ‘the other’ to DDP. As well as being context-dependent, TAU is case-dependent, with services attempting to respond to the individual – and changeable – needs of the family. This led therapists to surmise that any comparisons with DDP would be a non-specific TAU, commonly housed within CAMHS:

*I think we would be more likely to have a control group which was business as usual, if you like. Not every case that was the control group would be the same…you wouldn’t be comparing DDP with consultation, you might be comparing with consultation followed by some direct work with foster carers, followed by some work with the child and the foster carers. Another case might be moving from consultation and a very short piece of work with the foster carers to individual stuff with the children… it would be a variety of those things with the clinician making a judgment, consultation by consultation, and might need to shift into something else (Site E).*

Furthermore, it was felt that there was no ‘gold standard’ approach when treating MAPP within TAU. Variability across services was something that was seen as realistic and justifiable:

*I don’t think the gold standard service exists anyway and there is huge variability in there as well. I mean there are pockets of consistent approaches that go on within those specialist services and then the variability starts to increase ….people are doing their best (Site D).*

Some therapists felt that using a specific intervention as a control, had it been possible to identify one, would not reflect the fact that in ‘real life’ TAU is more often something like social work management of the case rather than a definable therapy. In this sense, therapists wanted the evidence - if in favour of DDP - to be useable in saying that DDP was better than what was currently happening in their services rather than one specific therapy that may not be utilised in other sites:

*I would like to be able to say to my managers that DDP is more effective than doing nothing with these children. And if we don’t have that sort of case management and things like that I won’t be able to say that. I will just say ‘well we are a bit better than child psychotherapy’ and they will say ‘well actually we will send them on to child psychotherapy’ (Site C).*

It is clear that a qualitative element in an exploratory trial is needed in order to carefully describe the pathways and models that exist within any sites chosen to be part of a trial. It should be noted, however, that although challenging for a trial, variability is not perceived as negative. Instead, it is seen as largely reflective of the reality of working within the context of complex problems and interventions, as well as responding to individual need [17].

In general, our findings suggest that 1) most participants see an eligible DDP population as potentially similar to other therapeutic CAMHS populations; 2) there is a lack of a ‘gold standard’ TAU, meaning that randomisation between DDP and a control intervention looks feasible and ethical.

### Measuring change

Participants were asked to reflect on the most important aspects of change during DDP therapy to inform our decision-making around adequate measurement in a DDP RCT. Two main themes emerged from this discussion, which need to be carefully considered in terms of the timing and multi-faceted nature of assessment; 1) that change needs to be about relationship change, placing parents/carers outcomes as central and 2) that change is multi-faceted and non-linear with progress as a complex journey with set-backs along the way.

#### Relationship-based change

Therapists commonly reflected that the first point of change has to be in relation to the parent/carers’ understanding and awareness of MAPP in their children. For some, this was an arduous task with slow progress and a substantial amount of work required to enhance parent/carer readiness for DDP (The issue of ‘readiness’).

This involved accepting that there is no ‘quick fix’ and that carers/parents are a pivotal driving force of change. It also involved an acceptance that problems will recur but that there are tools that DDP can provide that build parent/carer resilience and ability to cope. In this sense, measures have to be used that take into account the ‘changeability of change:’

*I have a little boy at the moment who has really moved from being stuck in rages when he was really worried to being able to cry and admit that he has got a worry. So that’s a massive step then you sort of think ‘oh well, you know, maybe we can finish DDP’ and then he went on a massive nose dive which seemed to be in response to the fact that he was adopted but he seems to be coming out of that nose dive. These children don’t get ‘cured’, but the extremities and the sort of resilience of the parents and of the child change. If he goes into another dip then they are much more likely to be able to think ‘oh what’s going on here, how come ....?’ They’ve now got a script (Site C).*

Parent/carers’ understanding of the problems and rationale for symptoms displayed was seen as the foundation for lasting change even if changes in the child’s behaviour were not always evident within the lifetime of DDP:

*The feedback I have from carers is that the behaviour doesn’t actually necessarily change but because they have a much more coherent peg to hang things on it helps them to get perspective and be more empathetic. That changes the interactions between everybody (Site A).*

#### Multi-faceted change

An overarching theme in relation to how DDP should be measured was the multi-faceted nature of change that results from DDP and the difficulty in finding assessment tools that can adequately reflect this change. For many, the goals of DDP are about working on the unique individual problems that families present with and assessing elements of change in relation to personal family goals. There were, however, some common outcomes of DDP identified and these included placement stability and a range of attachment-based behaviours as well as a reduction of behavioural symptoms. Longer-term change was regarded as the child being able to utilise the parent/carer and relationship to meet their needs:

*The things that concern managers are, is our looked after population stable in terms of placements? Ideally you would want to look at the child’s internal working models, the behaviour of the child in the relationship and symptoms which might indicate attachment-related problems; so behaviours that resist the closeness of the carer; aggression, conduct problems, oppositional problems and emotional regulation difficulties. Success in relation to DDP is when the child is open to the relationship when they have an attachment need; so they signal their needs clearly (Site C).*

Some elements of change were seen as small but significant and therapists reflected on how these aspects of progress are often difficult to pick up in assessment measures. It was reported that assessment of change needs to reflect this premise:

*So we are not going to expect that suddenly the child goes from nearly being excluded to being head boy…things like a child bringing an emotion into the room and being able to accept some sort of comfort from the carer – whether it is just a hand on the back – little steps that just represent a slight shift in the relationship would be incredibly encouraging and a more realistic aim (Site E).*

Therapists also talked about the importance of indices of change in the wider life context of the child that reflect how well the child is getting on with managing their relationships and wide-reaching implications for the future:

*Things like have they maintained relationships and friendships in school and out of school hobbies?….I think friendship is a very key one about how much resilience they might be developing. It’s these kind of measures for me (Site E).*

*What we want is for them to develop that self-regulation and safe base and from that the results could be amazing. You know, that could free you up to be able to concentrate better at school, to explore the world knowing that you have that safe base to go back to and it will affect relationships in the future and stress levels…to be able to support a child’s attachment style and increase their resilience would just be fantastic in all directions (Site C).*

## Conclusions

This study has demonstrated widespread interest, support and effective engagement regarding an RCT to explore the efficacy of DDP. The data suggests that there are a sufficient number of sites practicing DDP with the resources, managerial support and agreement from stakeholders to be involved in an exploratory trial. There are also some sites which require further time for development of DDP, which may become trial sites should circumstances develop or change whilst funding is sought. This gives us significant ‘back-up’ in terms of generating the numbers required to power a trial.

An exploratory RCT of DDP in the UK looks not only possible but also timely. We have been able to propose a potential trial model based on the findings of this study and in-depth qualitative investigation has generated a comprehensive picture of the ways in which DDP is operating across participating sites. This allows us to carefully consider aspects of DDP models that impact on the design of an RCT; particularly the need to clearly define what we mean by the therapeutic model of DDP, how DDP should be measured and the ways in which different pathways into a family receiving DDP can be managed within an RCT. Concurrently, we have been able to ascertain that a control intervention would be mainstream CAMHS and, given wide variability between sites, key point of investigation for an exploratory trial would be to describe, in detail, the nature of TAU contexts within sites.

The multiple points of engagement between the researchers and participants in this study enabled us to foster good relations with key stakeholders ahead of an RCT and we hope that this promotes a sense of partnership as well as ownership amongst sites in terms of the trial design. This feasibility study underscores the value of qualitative feasibility work in optimising the conditions for an exploratory RCT, particularly within the context of complex interventions [17] by uncovering important factors that impact on, and inform, the design of RCTs whilst also serving to engage potential participating centres.

## Abbreviations

ADHD: Attention deficit hyperactivity disorder
ASD: Autistic spectrum disorders
CBT: Cognitive behavioural therapy
DDP: Dyadic Developmental Psychotherapy
CAMHS: Child and adolescent mental health services
MAPP: Maltreatment associated psychiatric problems
NHS: National health service
PACE: Playfulness, acceptance, curiosity, empathy
NICE: National institute for health and clinical excellence
RCT: Randomised controlled trial
TAU: Treatment as Usual
UK: United Kingdom
US: United States
